# Comparison of Mesiodistal Root Angulation Measured from Conventional and CBCT Derived Panoramic Radiographs in Orthodontic Patients

**DOI:** 10.2174/1874210601711010338

**Published:** 2017-06-30

**Authors:** Ibrahim Nasseh, Douglas Jensen, Marcel Noujeim

**Affiliations:** 1 Department of DentoMaxillofacial Radiology and Imaging School of Dentistry, Lebanese University, Beirut, Lebanon; 2 Department of developmental dentistry The University of Texas Health Science Center, San Antonio School of Dentistry, San Antonio, TX, USA; 3 Oral and Maxillofacial Radiology The University of Texas Health Science Center, San Antonio School of Dentistry, San Antonio, TX, USA

**Keywords:** CBCT, Mesiodistal root, Panoramic radiographs, Conventional images, Pan-like images

## Abstract

**Introduction:**

Use of cone beam computed tomography (CBCT) in orthodontics is increasing; however, some patients started treatment with conventional images. The objective of this study is to manipulate CBCT panoramic reconstruction to make it comparable to conventional panoramic image and to compare mesiodistal root angulations on both images.

**Materials and Methods:**

Concurrent conventional panoramics and CBCT volumes were obtained from 40 subjects. CBCT volumes were manipulated to generate pan-like images that mimic the occlusal plane angle of the corresponding panoramic, allowing comparison of mesiodistal root angulations and determination of the head-tilt required to produce the reconstruction.

**Results:**

Clinically meaningful differences (*p* < .05) in the mesiodistal root angulations between standard panoramics and CBCT reconstructions emerged for 13 out of 24 teeth (54%). Greatest variations were seen in the maxillary and mandibular sextants and in first molar regions. Ideal axial head-tilt for image acquisition was determined to be with Frankfort horizontal plane 3.3^o^ nose down.

**Conclusion:**

CBCT pan images must be used with caution due to variation between methods in specific areas of arches. The images can be useful for the assessment of mesiodistal root angulations if the volume is properly manipulated to create a pan-like image.

## INTRODUCTION

During dental rehabilitation, whether it is by orthodontic or prosthodontic correction of malocclusion, the clinician is concerned with the teeth being oriented ideally within the bone in all three planes [1, 2]. For best functioning of the stomatognathic system, occlusal forces should be directed along the long axis of the teeth [3-5]. If ideal alignment is impossible due to unfavorable angulations of the teeth, the orthodontist may be called upon to move the teeth into a more biomechanically advantageous position, which will withstand occlusal forces and provide better stability. Executing such objectives requires a proper radiographic survey. The conventional panoramic radiograph (pan) serves as a diagnostic tool most often used to assess the teeth and their axial inclinations and to evaluate root parallelism prior to, during, and after orthodontic treatment [6, 7]. Despite their widespread use, conventional panoramics have been criticized for their dimensional inaccuracy [8-12]. The general agreement is that, although their accuracy is contested, that lack of precision does not affect most dental assessments. However, for clinical decisions that call for greater accuracy the pan images are considered less useful. Because the assessment of root angulation falls into this group, treatment decisions based on conventional panoramics should be made with caution. Not withstanding its inherent inaccuracy with respect to assessment of mesiodistal root angulation, the relative mesiodistal inclination of the roots of the maxillary and mandibular teeth, as judged from a panoramic radiograph remains an important assessment factor in the American Board of Orthodontics examinationas well as to clinicians in practice as a convenient pre, mid, and post-treatment diagnostic tool [6,13].

Recently, cone-beam computed tomography (CBCT) has been introduced as a great advancement in dental radiography. This technique provides a three-dimensional (3D) volume of data which can be rendered at any desired angle from a single imaging session. Several types of images, such as two-dimensional (2D) lateral cephalograms and panoramic views can be generated by manipulating the 3D volume data. The exactness of CBCT 3D images has been confirmed in a number of studies [14-19]. This technology can have great clinical implications for orthodontists, but especially if it can be demonstrated that the reconstructed 2D images portray dental angulations accurately enough to compare them to the preoperative panoramic images. If this is the case, clinicians could rely on CBCT images to confidently assess the alignment of mesiodistal tooth angulationswithout taking the linear measurements in consideration knowing that some studies proved a great correlation between these two techniques in linear measurement [20].

This study was based on the hypothesis that it is possible to derive 2D pan-like images from CBCT scanned data in such a way that direct side by side mesiodistal root angulation comparisons with conventional panoramics can be made. Furthermore, it was hypothesized that significant differences would be detected in the mesiodistal root angulation measurements between conventional pan and CBCT derived pan-like images. It was expected that the results of this study would determine a useful reference plane for positioning patients in the CBCT cephalostat, as well as provide an increased understanding of how mesiodistal root inclination assessment with the newer method varies compared to conventional pan radiography.

The specific aims of this study are:

1. To assist clinicians to generate 2-D pan-like reconstructions from CBCT volumes that arecomparable to traditional pan images.
2. To determine if there is a clinically significant difference between mesiodistal root angulations of teeth measured on conventional panoramics and CBCT derived pan-like reconstructions.

## MATERIALS AND METHODS

This retrospective, observational study was designed to allow the comparison of the mesiodistal root angulations on x-rays taken from subjects who had received both conventional panoramics and CBCT scans within the same time frame.

A *priori* statistical power analysis was performed assuming that a difference between imaging methods of 2.5˚ or greater is clinically meaningful [8, 9, 16]. With a minimum sample size of n=34 pairs of images; one CBCT and one panoramic image, a paired t-test was determined to have 80 percent power to detect a mean difference equal to 2.5˚, assuming a standard deviation of differences equal to 5 with a two-sided alternative hypothesis and a statistical significance defined as *p* < .05 [17, 18].

Forty suitable study subjects were obtained from the data base of the University of Texas Health Science Center San Antonio School of dentistry. Eighteen males and twenty-two females with the average age of 15 years and a standard deviation of ± 3.5 years. Acquisition of CBCT volumes is not a routine practice in orthodontics department but these volumes were acquired for multiple purposes including, and not limited to, determination of positions of impacted canines and third molars, TMJ issues, trauma and few pathology cases. Panoramic images were acquired with the Planmeca Proline (Helsinki, Finland)using the standard settings for young patients (7mA and 80 kVp) and CBCT volumes with the Planmeca ProMax® 3D Midequipped with the Planmeca Ultra Low Dose™ protocol (5.6 mA, 90 kVp and 4 sec.). The study was approved by the institutional review board of the health science center. Data collection duration was for four weeks, image processing, measurements and statistical analysis duration was six months.

The inclusion criterion was the presence of concurrent panoramic and CBCT scans taken with acceptable head orientation and image resolution. Acceptable head orientation is defined by a slightly concave occlusal plane on the panoramic views, images with a flat or convex occlusal planes were excluded.The images were assessed based on occlusal plane angle (representing an appropriate axial head tilt) and the lack of left/right head rotation. Images were also evaluated for adequate contrast and resolution to enable precise measurement. All panoramic images were 8 bit and 300 dpi or better, CBCT volumes had 0.3 mm voxel size maximum. Only missing teeth or primary teeth were excluded from measurement if panoramics were otherwise readable. Each subject was assigned a random identification number so that the primary investigator would be masked to the subject when measuring the pan radiographs and CBCT scans. The available permanent teeth in the maxilla and mandible from first molar to first molar were evaluated on the pan and pan-like images pairs of n = 40 subjects.

**Fig. (4):**
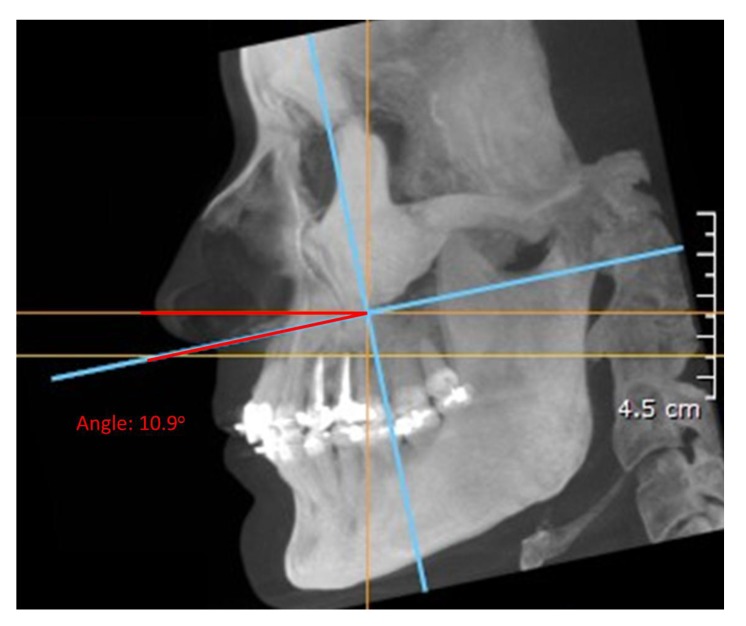
Cephalometric view showing angle between initial and final orientations of CBCT volume.

As a reference for convenient and accurate angle measurement of the teeth, the occlusal plane was established in close proximity to the teeth being measured [16]. To prepare the pan and CBCT pan-like image pairs for measurement, the occlusal plane of each subject was determined by establishing bilaterally the midpoints of the incisal edge of the central incisors and extending lines to intersect the buccal cusp tips of the first molars bilaterally [17]. The angle formed by the intersection of these two occlusal lines was measured and the resultant angle formed the basis for the comparison of the pan and CBCT pan-like radiographs. Next, the corresponding CBCT scans obtained in DICOM (digital imaging and communication in medicine) were imported into CBCT file manipulating software On Demand 3D (Cyber med Inc. South Korea), and a two-dimensional pan-like view of the volumetric data was created. The panoramic reconstruction was made by plotting a series of points around the arch form and back through the mandibular rami, the curve was parallel to the maxillary and mandibular posterior teeth, and slightly anterior to the mandibular incisors Fig. (**1**). The axial slice thickness for creating the image was set at approximately 25 mm. The occlusal plane angle on the resultant pan-like image was determined with the same technique as for panoramics, and compared to the conventional pan image. If the two angles did not initially coincide, a volume reorienting feature within On Demand software was used to tilt the head up or down as needed to bring the plane angles into agreement within two degrees of each other Figs. (**2** and **3**). To quantify the changes with regard to the horizon, a cephalometric view of the volume enabled the plotting of the Frankfort horizontal reference plane for each subject and this plane was used to determine the corresponding change in axial orientation up (a positive change) or down (a negative change) for each pan-like image. Approximate head orientation used during exposure of the conventional pan radiograph was therefore deduced. The change to axial head tilt for each subject was measured and logged for analysis (Fig. **4**).

The mesiodistal root angulations of the teeth from first molars anteriorly in all quadrants on conventional panoramics were determined relative to the occlusal plane established using ImageJ, a public domain image measuring software (Wayne Rasband, Maryland, USA). The long axis of single-rooted teeth was represented by a line drawn from the midpoint between CEJ’s at the coronal aspect through a point at the root apex. The long axis of multi-rooted teeth was determined by drawing a line from the midpoint between CEJs at the cervix to the midpoint between the most mesial and distal root apices [18]. A mesiodistal inclination value greater than 90˚ indicated a distal inclination to the root, and a value less than 90˚ indicated a mesial inclination to the root [17]. Lastly, the corresponding pan-like CBCT images were converted to JPEG images while maintaining the initial aspect ratio of the x-rays, lines representing the long axes of the teeth were drawn, and mesiodistal root angles were measured by hand using 3M Unitek’s cepahometric protractor (Figs. **5A**-**B**).

Prior to the initiation of data collection, we assessed data for a sample of 5 subjects on two occasions 2 weeks apart, using the same methods used in the study proper. Intra-rater reliability was estimated by calculating intra-class correlation coefficients. This analysis showed that the measurement of root angles was highly repeatable.

### Statistical Analysis

Mean adjustments to the axial head tilt were calculated. Descriptive statistics, including mean, standard deviation and 95% confidence intervals of root angulation were calculated for the difference between imaging methods. A linear mixed models approach (IBM SPSS version 19, Armonk, NY) was used to test the null hypothesis that root angulations measured on pan and CBCT pan-like radiographs are not different. This two-factor, completely within subjects analysis assessed the main effects of measurement method (2 levels) and tooth (24 levels) and their interaction. Following significant results in the omnibus analysis, the two methods were compared for each tooth using Fisher’s least significant difference procedure, with all null hypotheses rejected at the two-sided *p* < .05 significance level. Bonferroni corrected p-values also were calculated to guard against type I statistical errors (false positives).

## RESULTS

In order to make the two imaging methods comparable, CBCT volumes required changes to the axial head tilt prior to root angle measurements which ranged from +9˚ to -16˚. Twenty-nine out of 40 (72.5%) of subjects required a negative adjustment (nose down) to make the CBCT pan-like image agree with the standard pan, while 9 of 40 subjects required a positive adjustment (nose up). Only 2 subjects (5%) required no change due to their initial agreement. Mean adjustment value of the axial head tilt was -4.6˚, indicating that most CBCT scans are taken with a nose up head tilt, with Frankfort horizontal plane not being parallel to the true horizontal (relative to the average well-taken conventional pan). Reducing the axial head tilt an average of -4.6˚ resulted in an average Frankfort horizontal to true horizontal measurement of -3.3˚. Table (**1**) shows the occlusal plane angle measurements, axial head tilt changes, and resultant Frankfort horizontal to true horizontal measurements of the 40 subjects.

In the omnibus analysis of the measured tooth pairs, all main and interaction effects were statistically significant at the *p*< .001 level. The main effect of imaging method was significant [F(1,39) = 37.88, *p* < .001], indicating that the measured root angles obtained under the two methods, pan versus pan-like images were different when averaging over all 24 teeth (mean for CBCT = 97.21˚ versus 95.06˚ for pan). The main effect of tooth [ F(23,118) = 23.64. *p* < .001] was also significant indicating that root angle depends on which tooth in the arch. Furthermore, the interaction effect between imaging method and tooth was significant [ F(23,121) = 5,84, *p* < .001), demonstrating that the difference between methods depends on which tooth is being considered. Therefore, the difference between methods was further studied by comparing CBCT pan-like images versus panoramics for each tooth. Table (**2**) displays the mean differences, standard deviations, and 95% confidence intervals for the differences between root angles derived from pan and pan-like images for each tooth.

Compared to standard panoramics, measurements from pan-like images were not significantly different at the uncorrected *p*< .05 level for maxillary teeth 11-15 and 24-26. In the mandible, only three teeth 35, 45, and 31 were not significantly different. All other mandibular teeth, particularly first premolar to first premolar (except 31) showed significant differences. Additionally, pan-like images tended to depict the maxillary roots from teeth 15-25 more distally and the mandibular roots from 34-45 more distally. The remaining posterior teeth, except for tooth 45, were depicted with mesial inclination relative to standard pan angulations.

The more notable differences between methods were found for the upper left anterior region with teeth 22 (4.1˚) and 23 (5.1˚). Stark differences were found especially in the mandible where, except for tooth 31, teeth 34-44 differed from 4.1˚ to 8.9˚. Not only were the differences highly significant, but the corresponding standard deviations were larger for these teeth as well. Table **3** provides a graphic for angle measurement comparisons, where larger variation exists, and whether there is a mesial or distal tendency to the angle projection by CBCT relative to standard panoramics. Employing the Bonferroni correction to the p-value of each tooth, we multiplied each p-value by 24 (the number of teeth). Even by this highly conservative standard, statistically significant differences persisted for several teeth (*i.e.,* 23, 32-36, and 41-44). The Bonferroni *p*-values are presented in (Table **2**).

## DISCUSSION

The results relate only to the panoramic unit used in this study (Planmeca Proline) and the use of the "On demand 3D software" for manipulation of the DICOM files. However, it has been shown that several panoramic units are similar in the projection of mesiodistal root angulations [16].The consensus from several studies is that standard panoramics taken by various panoramic machines, including the Planmeca Proline used in the present study, have the tendency to project maxillary anterior roots slightly more mesially and posterior roots more distally than true angles found on typodonts. In the mandible nearly all roots are projected more mesially than they actually are. The larger angular differences in the maxilla occur between the canines and first premolars. The larger angular differences between adjacent teeth in the mandible occur between the mandibular lateral incisors and the canine, with relative root parallelism projected as root convergence [9, 16, 17]. These limitations should be well understood when assessing root angulations or considering standard panoramics for comparison.

The purpose of this study was to determine a useful reference plane for positioning patients in the CBCT cephalostat, to be able to generate images that can be used to compare to panoramic images taken previously as well as provide an increased understanding of how mesiodistal root inclination assessment with the newer method varies compared to conventional pan radiography.

In order to create pan-like images that are comparable for this study, CBCT DICOM files must be manipulated via computer software in such a way as to produce a 2D view of a 3D object. During the course of this study, it was observed that the ability to produce a high-resolution 2D image free from obvious root distortions varied depending on the axial orientation of the volume used and on the method of plotting points along the arches. Image integrity was apparently lost particularly along the line of the tightest curve of the anterior mandible. This may account in part for why the greatest differences between imaging methods occurred in the anterior mandible. Refined operator technique to some extent, and further technological improvement in software rendering inevitably should improve image quality during software manipulation of the 3D volume into a 2D pan and reduce the resultant distortions.

This study included 40 subjects, with possibly none exhibiting ideal occlusion. Although the inclusion criteria for the study did not include an assessment of each subject’s occlusion, many subjects were of orthodontic age and contemplating orthodontic treatment who presumably exhibited a degree of malocclusion. Although from an assessment of the 3D volume, it might have been possible to determine the degree to which the included subjects in the study presented with various features associated with malocclusions, it was only noted that features existed. The presence of such things as constricted arch forms, crowding in the maxilla and mandible, acute inter-incisal angles, and unusual occlusal plane angles seemed to influence the observer’s ability to obtain distortion-free pan-like images. Through the trial and error process of creating pan-like images, it was found that these various features, in addition to the previously mentioned axial head tilt and plotting of points for generating the 2D view, also seemed to influence the resolution of the images, the degree of distortions, and ultimately the mesiodistal angulations of the teeth in the anterior sextants.

Notwithstanding the presence of features that influence the generation of a pan-like image, the best subjective depiction of the anterior teeth was obtained when the axial head tilt corresponded to Frankfort horizontal being about 3-4 degrees nose down from the true horizon. This is helpful to know when positioning the patient’s head in the cephalostat to preclude the need to reorient the 3D volume in the software prior to creating a 2D pan-like image, thereby decreasing the time it takes to create the image. It is suggested that clinicians or technicians performing the scans keep this head position in mind as a good position for minimizing post-scan rendering effort. However, if the scan happened to be taken with the head oriented too high or low, this recommended head orientation serves as a point to begin creation of the pan image within the software. Further research directed toward the determination of the ideal orientation of the patient’s occlusal plane from the cephalometric perspective relative to true horizontal would be most appropriate for reducing distortions in pan-like images. Although Frankfort horizontal (Po-Or) plane is convenient due to readily discernible external landmarks, it is the ideal orientation of the occlusal plane that would give rise to the greatest consistency in the creation of pan-like images for analysis of mesiodistal root angulations. A study determined the optimal orientation of the occlusal plane would also be informative.

A pattern of statistically significant differences in the mesiodistal root angulations between standard panoramics and CBCT pan-like images emerged for 13 out of 24 teeth (54%). All first molars except for tooth 26, the upper left anterior (teeth 21-23), and the lower teeth from first premolar to first premolar, except for tooth 31 were significantly different. The magnitude of the differences in mesiodistal angles measured relative to the occlusal plane was larger in the mandible from first premolar to first premolar. If a tolerance limit of ±2.5˚ is applied, there remained statistically *and* clinically significant differences in measured root angulations in the mandibular anterior sextant [8, 9, 16]. The greatest standard deviations were found in the maxillary and mandibular anterior regions of the arches indicating where imaging methods vary the greatest.

Obviously, it is not a requirement to use CBCT pan-like image to assess mesiodistal root angulations. Other methods exist; Bouwens *et al* explored an alternative method in the use of *in vivo* Dental 3D software to create custom sections from axial slices, with measurements of each tooth made from a facial view relative to the occlusal plane [19]. Ideal use of the CBCT volume would come, not from the use of a pan-like image, but instead by a study of the entire 3D volume within the volume-rendering mode contained within most imaging software. It has been shown that this view is dimensionally accurate and one could be assured of realistic assessment of root angulation [15, 20, 21]. The question of convenience arises in this case due to the requirements to obtain such renderings. Rather than having a technician, an auxiliary, or the doctor creating the pan-like image and saving it in management software, an assessment of the full volume requires real-time loading of the DICOM files into the viewing software on a computer with demanding processing requirements. Assessment would thus be more time consuming and hardware dependent. Therefore, time and cost constraints may be factors in the use of this technology, reducing the prevalence of its use. Although CBCT has much higher potential for a complete assessment of the craniofacial complex, if one understands the variation inherent in standard panoramic radiology, this method may remain the more accurate, convenient, and clinically practical method for assessment of mesiodistal root angulation.

The significance of the study is its ability to provide a method for acquiring CBCT volumes able to generate panoramic reconstructions that can be compared to pre- or post-orthodontic treatmentpanoramic images taken prior or after a CBCT volume.

## CONCLUSION

The following conclusions can be drawn from this study:

1. CBCT-derived pan-like images can be made comparable to that of conventionally taken panoramic radiographs, allowing direct comparison of mesiodistal root angles from both types of images. To create pan-like images that resemble the conventional pan images, the patient’s head should be positioned with Frankfort horizontal plane oriented 3.3 degrees nose down to true horizontal. Alternatively, the 3D volume could be oriented within the software with the same orientation as a starting point for deriving the pan-like image.
2. Greatest differences in the mesiodistal root angles between the pan and CBCT pan-like images were observed in the upper and lower anterior sextants, and in the first molar areas. CBCT pan-like images are less consistent than standard panoramics in their depiction of mesiodistal root angulation, especially in the maxillary and mandibular anterior regions, as well as in molar regions, and must be used with an understanding of the potential root distortion that occurs when generating these images. However, the images can be a useful adjunct for assessment of mesiodistal root angulations if the clinician is knowledgeable about its limitations and how the volume is manipulated in creating a pan-like image.
3. A pattern existed with the CBCT scan depicting from maxillary first premolar to first premolar with a more distal angulation compared with standard panoramics, and second premolars and molars with a more mesial angulation. In the mandible, a similar pattern emerged, where the lower right second premolar to lower left first premolar was depicted more distally, and the rest of the posterior teeth were more mesial inclined.

## Figures and Tables

**Fig. (1):**
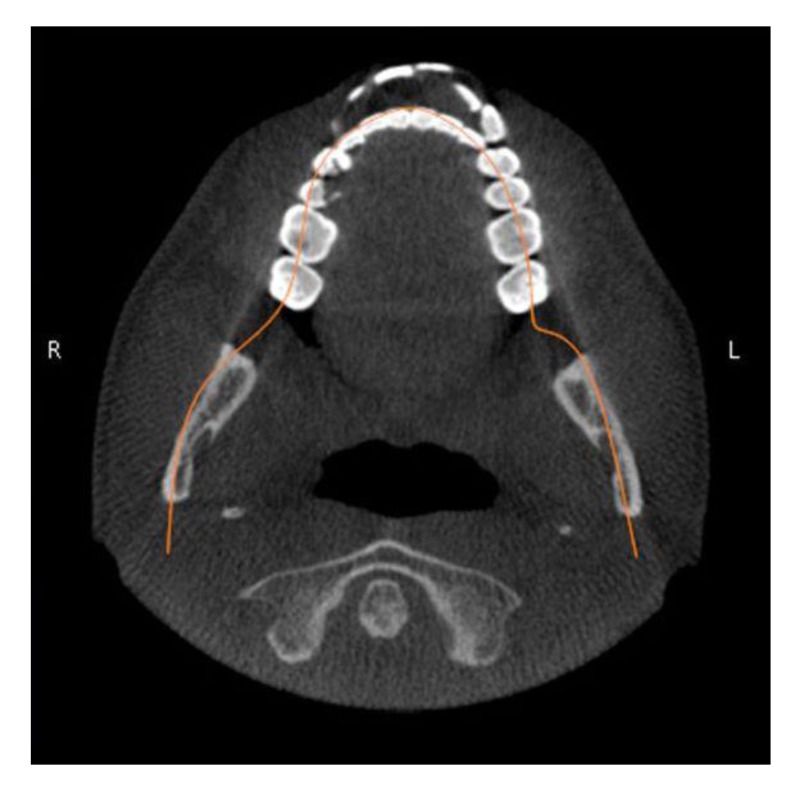
Tracing a guide line to generate the panoramic reconstruction.

**Fig. (2):**
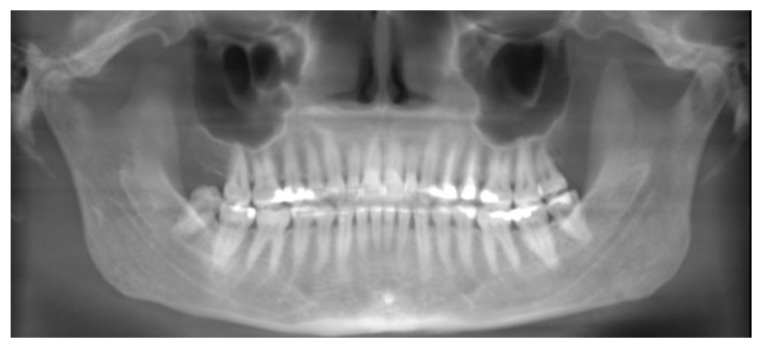
Pan-like reconstruction from CBCT volume at initial head orientation.

**Fig. (3):**
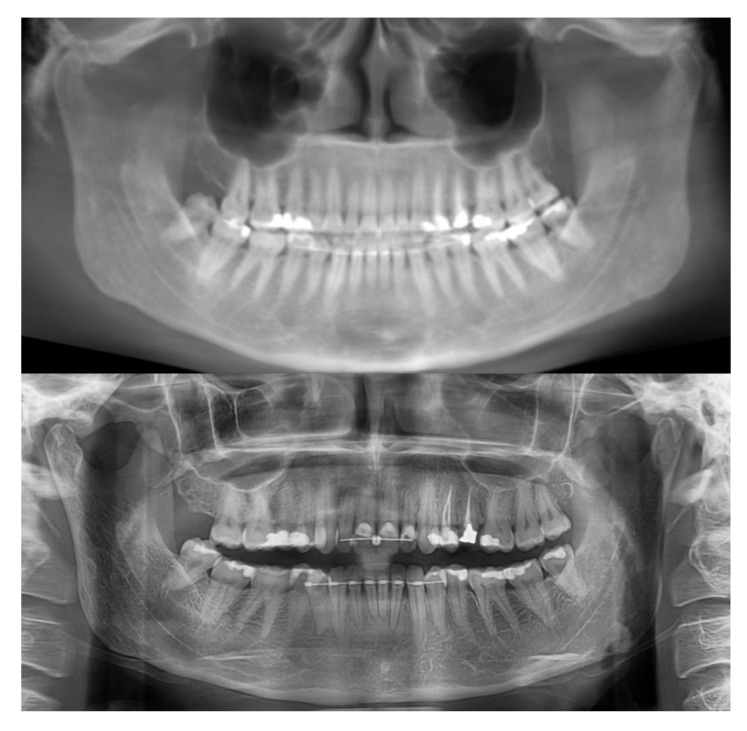
Pan-like reconstruction from CBCT volume after axial re-orientation and creating agreement with conventional pan.

**Fig. (5A):**
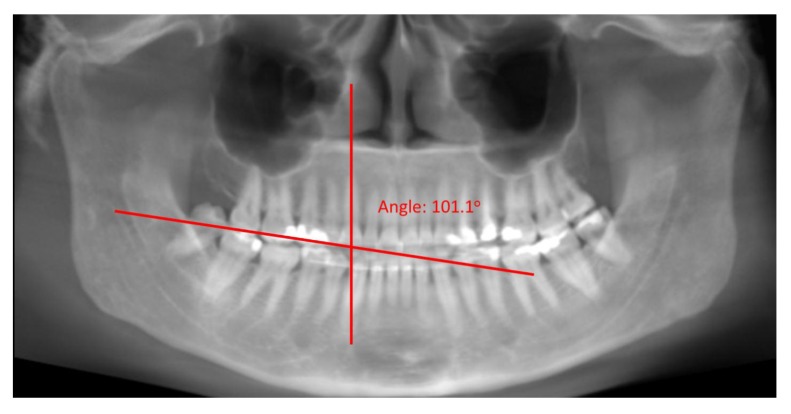
Occlusal plane established and angle between long axis of tooth and occlusal plane measured showing a distal (A) and mesial (B) inclinations of the root.

**Fig. (5B):**
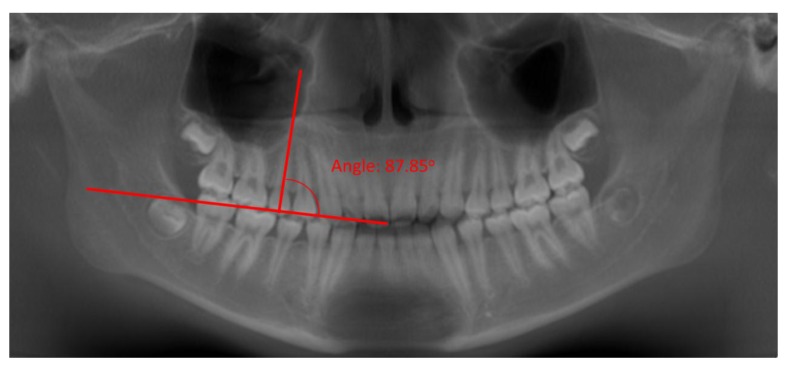
Occlusal plane established and angle between long axis of tooth and occlusal plane measured showing a distal (A) and mesial (B) inclinations of the root.

**Table 1:** Initial and adjusted occlusal plane and Frankfort plane angle values.

**Subject**	**Pano Occlusal Angle (Deg)**	**Adjusted CT Pan-like Occlusal Angle (Deg)**	**Initial Frankfort Plane to Horizon (Deg)**	**Axis Change (Deg)**	**New Frankfort to Horizon (Deg)**
1	158.9	158.2	6.0	-6.0	0.0
2	161.6	159.9	0.0	0.0	0.0
3	145.6	144.8	0.0	-2.0	-2.0
4	159.8	159.5	14.5	-16.0	-1.5
5	163.6	162.8	3.5	-9.5	-6.0
6	156.4	157.6	7.0	-11.5	-4.5
7	159.3	160.7	-4.5	-1.5	-6.0
8	161.7	160.8	8.0	-14.5	-6.5
9	160.1	160.7	1.0	2.5	3.5
10	163.4	161.7	2.5	-1.5	1.0
11	162.7	164.3	0.0	-6.5	-6.5
12	172.1	171.9	8.0	-6.0	2.0
13	156.6	155.0	5.5	-15.0	-9.5
14	165.4	163.5	0.0	-9.5	-9.5
15	171.5	170.6	7.0	0.0	7.0
16	157.2	157.0	3.5	-8.0	-4.5
17	164.3	162.5	14.0	-14.0	0.0
18	164.2	164.3	3.5	-7.5	-4.0
19	157.3	157.6	-4.5	-5.0	-9.5
20	172.0	172.6	4.5	-2.5	2.0
21	157.4	156.0	0.0	-8.0	-8.0
22	164.0	166.0	0.0	-2.0	-2.0
23	154.7	155.0	4.5	-12.0	-7.5
24	162.2	162.4	0.0	-2.0	-2.0
25	159.5	160.0	-1.0	-4.0	-5.0
26	150.9	150.0	-4.5	-10.0	-14.5
27	165.0	165.3	-2.0	1.5	-0.5
28	154.1	153.9	2.5	-5.5	-3.0
29	152.5	153.9	-2.0	-10.0	-12.0
30	169.1	169.2	-5.0	2.0	-3.0
31	154.8	153.6	-12.0	3.0	-9.0
32	175.7	176.0	-9.5	6.5	-3.0
33	163.1	163.4	-4.5	4.5	0.0
34	170.7	172.0	1.5	0.5	2.0
35	157.5	157.4	-10.5	4.0	-6.5
36	162.3	162.1	9.0	-10.0	-1.0
37	150.1	150.3	-4.5	-1.5	-6.0
38	157.4	157.2	8.5	-10.5	-2.0
39	165.4	165.4	3.5	-4.0	-1.5
40	171.8	170.4	-3.0	9.0	6.0
**Mean**	161.3	161.1	1.3	-4.6	-3.3

**Table 2:** Mean differences between mesiodistal angles of conventional pan and CBCT pan-like images (n = 40).

Tooth Number*		Mean Difference (˚)	SD	95% CI for Difference	*p*-value	Bonferroni *p*-value
	16	-3.2	3.9	-5.832, -.568	.017	.408
	15	-0.4	4.8	-3.017, 2.247	.774	1.00
	14	+0.4	5.4	-2.268, 3.035	.777	1.00
	13	+.09	4.6	-1.734, 3.529	.503	1.00
	12	+2.4	9.3	-.222, 5.042	.073	1.00
	11	+0.9	6.9	-1.684, 3.579	.480	1.00
	21	+2.7	6.0	.068, 5.332	.044	1.00
	22	+4.1	7.9	1.431, 6.694	.003	.072
	23 **	+5.1	5.8	2.250, 7.555	.000	.000
	24	+0.6	4.9	-2.049, 3.257	.655	1.00
	25	-0.8	5.1	-3.374, 1.932	.594	1.00
	26	-2.5	5.2	-5.147, .117	.061	1.00
	36 **	-4.7	6.6	-7.317, -2.053	.001	.024
	35	-0.7	5.5	-3.312, 1.952	.612	1.00
	34 **	+5.6	8.8	2.948, 8.212	.000	.000
	33 **	+8.3	11.6	5.621, 10.884	.000	.000
	32 **	+8.9	16.1	6.261, 11.524	.000	.000
	31	+1.9	14.3	-.722, 4.542	.155	1.00
	41 **	+6.4	11.6	3.761, 9.024	.000	.000
	42 **	+8.0	13.0	5.336, 10.599	.000	.000
	43 **	+7.2	9.9	4.548, 9.812	.000	.000
	44 **	+4.1	7.9	1.501, 6.764	.002	.048
	45	+.3	5.8	-2.397, 2.907	.850	1.00
	46	-3.7	6.3	-6.199, -.894	.009	.216

* FDI tooth numbers.

Mean difference = (CBCT mesiodistal angle) - (standard pano mesiodistal angle) *P* < .05 is considered statistically significant.

** Indicates teeth measurements remain statistically significantly different after Bonferroni *p*-value adjustment.

**Table 3:** Graphic for M-D root angle measurement comparisons: CBCT *vs* Pano.

		**UR**											**UL**
**Correlation**		.890	.858	.902	.923	.653	.521	.692	.637	.769	.777	.816	.858
***p* value =**		.017	.774	.777	.503	.073	.480	.044	.003	.000	.655	.594	.061
**Tend for M/D root tip**		M	M	D	D	D	D	D	D	D	D	M	M
**Mean difference, deg**		-3.2	-0.4	+0.4	+0.9	+2.4	+0.9	+2.7	+4.1	+5.1	+0.6	-0.8	-2.5
Tooth #*		**16**	**15**	**14**	**13**	**12**	**11**	**21**	**22**	**23**	**24**	**25**	**26**
Tooth #*		**46**	**45**	**44**	**43**	**42**	**41**	**31**	**32**	**33**	**34**	**35**	**36**
**Mean difference, deg**		-3.7	+0.3	+4.1	+7.2	+8.0	+6.4	+1.9	+8.9	+8.3	+5.6	-0.7	-4.7
**Tend for M/D root tip**		M	D	D	D	D	D	D	D	D	D	M	M
***p* value =**		.009	.850	.002	.000	.000	.000	.155	.000	.000	.000	.612	.001
**Correlation**		.690	.682	.562	.654	.615	.240	.390	.462	.624	.564	.702	.666
		**LR**											**LR**
		* FDI numbering system											
		Mean difference = (CBCT mesiodistal angle) - (standard pano mesiodistal angle)											
		Mean difference in degrees between CBCT/Pano tooth pairs : (-) = CBCT mean angle is lower– indicating more mesial root tip											
							(+) = CBCT mean angle is higher—indicating more distal root tip						
		Shaded: Highlights where mean difference between pano and CBCT root angles are significantly different at *p*< .05.											
		*P*< .05											
